# Depth-Independent Reproduction in the Reef Coral *Porites astreoides* from Shallow to Mesophotic Zones

**DOI:** 10.1371/journal.pone.0146068

**Published:** 2016-01-20

**Authors:** Daniel M. Holstein, Tyler B. Smith, Claire B. Paris

**Affiliations:** 1 Marine Biology and Fisheries, Rosenstiel School of Marine and Atmospheric Science, University of Miami, Miami, Florida, United States of America; 2 Center for Marine and Environmental Studies, University of the Virgin Islands, St. Thomas, United States Virgin Islands, United States of America; 3 Department of Ocean Sciences, Rosenstiel School of Marine and Atmospheric Science, University of Miami, Miami, Florida, United States of America; U.S. Geological Survey, UNITED STATES

## Abstract

Mesophotic coral ecosystems between 30–150 m may be important refugia habitat for coral reefs and associated benthic communities from climate change and coastal development. However, reduced light at mesophotic depths may present an energetic challenge to the successful reproduction of light-dependent coral organisms, and limit this refugia potential. Here, the relationship of depth and fecundity was investigated in a brooding depth-generalist scleractinian coral, *Porites astreoides* from 5–37 m in the U.S. Virgin Islands (USVI) using paraffin tissue histology. Despite a trend of increasing planulae production with depth, no significant differences were found in mean peak planulae density between shallow, mid-depth and mesophotic sites. Differential planulae production over depth is thus controlled by *P*. *astreoides* coral cover, which peaks at 10 m and ~35 m in the USVI. These results suggest that mesophotic ecosystems are reproductive refuge for *P*. *astreoides* in the USVI, and may behave as refugia for *P*. *astreoides* metapopulations providing that vertical larval exchanges are viable.

## Introduction

Increasingly, the persistence of coral reefs may depend on populations of associated benthic species living in isolation or at the margins of their biogeographic ranges. Depth has been posited as a potential refuge for coral reef species from habitat degradation and climate change [1,2]. Because mesophotic coral ecosystems (MCEs) that form between 30–150 m depth can be remarkable in total area [1,3,4] and coral cover [5,6], the depth-fecundity relationships of depth-generalist species may have sweeping implications for the roles deeper populations play in population connectivity and as refugia from coastal development and climate change [7,8]. However, depth refugia may be limited with increasing depth. There is evidence of genetic differentiation over depth gradients in some species [9,10], and taxonomic breaks have been observed in benthic communities above and below 60 m [11]. Decreased photosynthetically active radiation may contribute to changes in photosynthate translocation from symbionts [12,13], and decreased tissue thickness [14] indicates that some scleractinian corals are limited energetically at the lower end of their depth ranges. Lower available energy can lead to decreased growth rates with increasing depth [15,16] which reduces the probability of transitions to larger size classes in deeper coral populations [17].

Energetic limitation for scleractinian corals growing at depth may curtail the reproductive performance of deeper living coral populations, which would restrict the potential for deeper, mesophotic habitat to supply larvae to other populations. Three brooding species from the Pacific (*Acropora palifera*, [18]; *Pocillopora damicornis*, [19]) and the Red Sea (*Stylophora pistilata* [20]) suggest a diversity of coral reproductive changes with depth. *Acropora palifera* and *S*. *pistilata* both produced fewer larvae with increasing depth, whereas no depth relationship was found for planulation in *P*. *damicornis*. A recent study of a spawning acroporid species that appears to be an upper mesophotic specialist, *A*. *tenella*, in Okinawa, Japan, suggests mesophotic individuals and populations are fully reproductive, although less fecund than shallow acroporids [21]. In contrast, mesophotic individuals of the broadcast spawning Caribbean coral *Orbicella faveolata* appear to outperform shallower conspecifics in egg production, and mesophotic populations may contribute more significantly to regional larval pools [8]. This may suggest acclimation or adaptation of some depth-generalist corals to reduced light. The depth-fecundity relationships of other Caribbean coral species are not well examined. The objective of this study was to establish the effects of depth on the reproduction of the common Caribbean scleractinian coral, *Porites astreoides*. This species is a brooding simultaneous hermaphrodite that occurs from 0.2 m to as deep as 70 m [22]. The coral is common both in shallow and mesophotic habitats in the USVI [5,23,24]. *P*. *astreoides* colonies can release gametes and fully developed planula larvae from April until as late in the year as September, with peak larval release focused around new moons in April and May [25–27]. Although the brooding time is not well documented, it may be as short as two weeks, the time between sperm release, around full moon, and planulation, around new moon [26]. We have observed common settlement and growth of *P*. *astreoides* juveniles at mesophotic depths (30–50 m), suggesting that this species can reproduce effectively over most of its depth range. For this reason, we hypothesized that reproduction and larval output of *P*. *astreoides* is not affected by depth across a gradient of 5–37 m.

## Materials and Methods

### Study area

**Fig 1:**
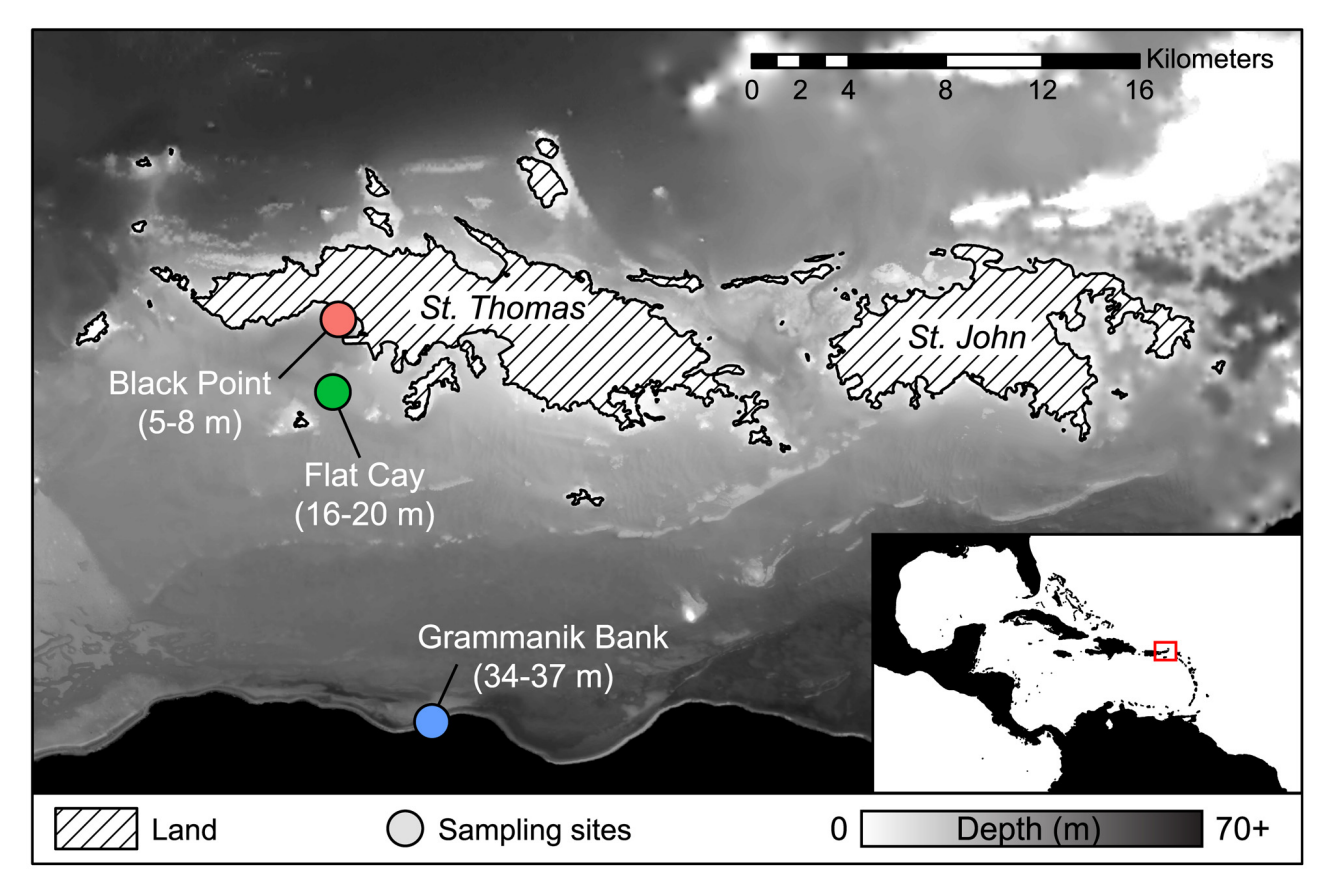
The northern US Virgin Islands of St. Thomas and St. John. Considerable mesophotic habitat (30–150 m) exists on the broad insular platform, and well-mapped linear coral habitat exists on submerged banks near the shelf edge south of St. Thomas [4,5,23]. Sample sites (circles) were visited weekly for five weeks in April-May. The British Virgin Islands of Tortola and Jost van Dyke are not shown.

The islands of St. Thomas and St. John make up the northern USVI, and are joined together and to the British Virgin Islands and Puerto Rico by the shallow Puerto Rican Shelf platform. The insular shelf south of St. Thomas is a gradual slope, and extensive linear MCEs can be found on submerged banks along the shelf edge [4] (Fig 1).

### Histological sampling and preparation

Tissue samples of *P*. *astreoides* (brown morph) were taken from shallow (Black Point, 5–8 m), mid-depth (Flat Key, 16–20 m) and mesophotic (Grammanic Bank, 34–37 m) coral colonies from reefs south of the island of St. Thomas in the United States Virgin Islands (USVI) weekly for five consecutive weeks April-May (2011). From each site in each week 5–8 samples were taken. A total of 76 samples yielded usable histological results, roughly equally distributed among sites and weeks (see S1 Dataset). Samples were collected under research permits F/SER28:BT (United States Department of Commerce, National Oceanic and Atmospheric Administration, National Marine Fisheries Service) and STT-021-10 (Government of the Virgin Islands of the United States, Department of Planning and Natural Resources, Division of Fish and Wildlife).

Tissue samples (~20 cm^2^) were taken from central high-points on colonies at least 100 cm^2^ using a hammer and cold chisel. All tissues were fixed in zinc-buffered formalin (Z-Fix) for 24 hours, and rinsed in fresh water for another 24 hours before being stored in 70% EtOH until histological processing. Samples were processed for paraffin histology according to the methods of Holstein et al. [8].

The longest diameter, perpendicular diameter and height were recorded for each sampled coral colony. Colony surface area was estimated as half the surface area of a three-dimensional scalene ellipsoid using the longest diameter, perpendicular diameter and the height of the colony [8,28]. This method should theoretically be a better estimator of coral surface area than a two-dimensional ellipse or an average hemisphere, particularly when making comparisons between shallow and mesophotic coral colonies that may change in morphology over depth.

*P*. *astreoides* histological sections were evaluated for (1) presence/absence of male and female gonads and planulae larvae, and (2) the density of larvae in the tissue per cm^2^. Slides were analyzed using both standard light microscopy as well as an Olympus VS120-S5 digital slide scanner, and measurements were made using the Fiji software package [29]. Fecundity was estimated as the number of planulae per cm^2^ of tissue on histological slides.

To derive depth-specific unit reef larvae production, estimates of percent coral cover were calculated from diver transects performed at each site by University of the Virgin Islands researchers from 2001–2012 [30]. *P*. *astreoides* fecundity was then multiplied by *P*. *astreoides* coral cover to estimate the number of larvae per 1 km^2^ unit reef as a function of depth:

$$Production\;=fecundity\;*\;{area}^{-1}\;*\;coral\;cover$$ (1)

### Aquarium observations

Three full *P*. *astreoides* colonies from each site (> 100 cm^2^, N = 9) were also brought to the lab and kept in shaded outdoor flow-through seawater tables for planulation observations (collected shallow on 4/27/2011; mid-depth on 4/29/2011; mesophotic on 5/2/2011). Observations were made on the evenings of May 2^nd^ and 3^rd^, 2011.

## Results

### Reproductive histology

**Fig 2:**
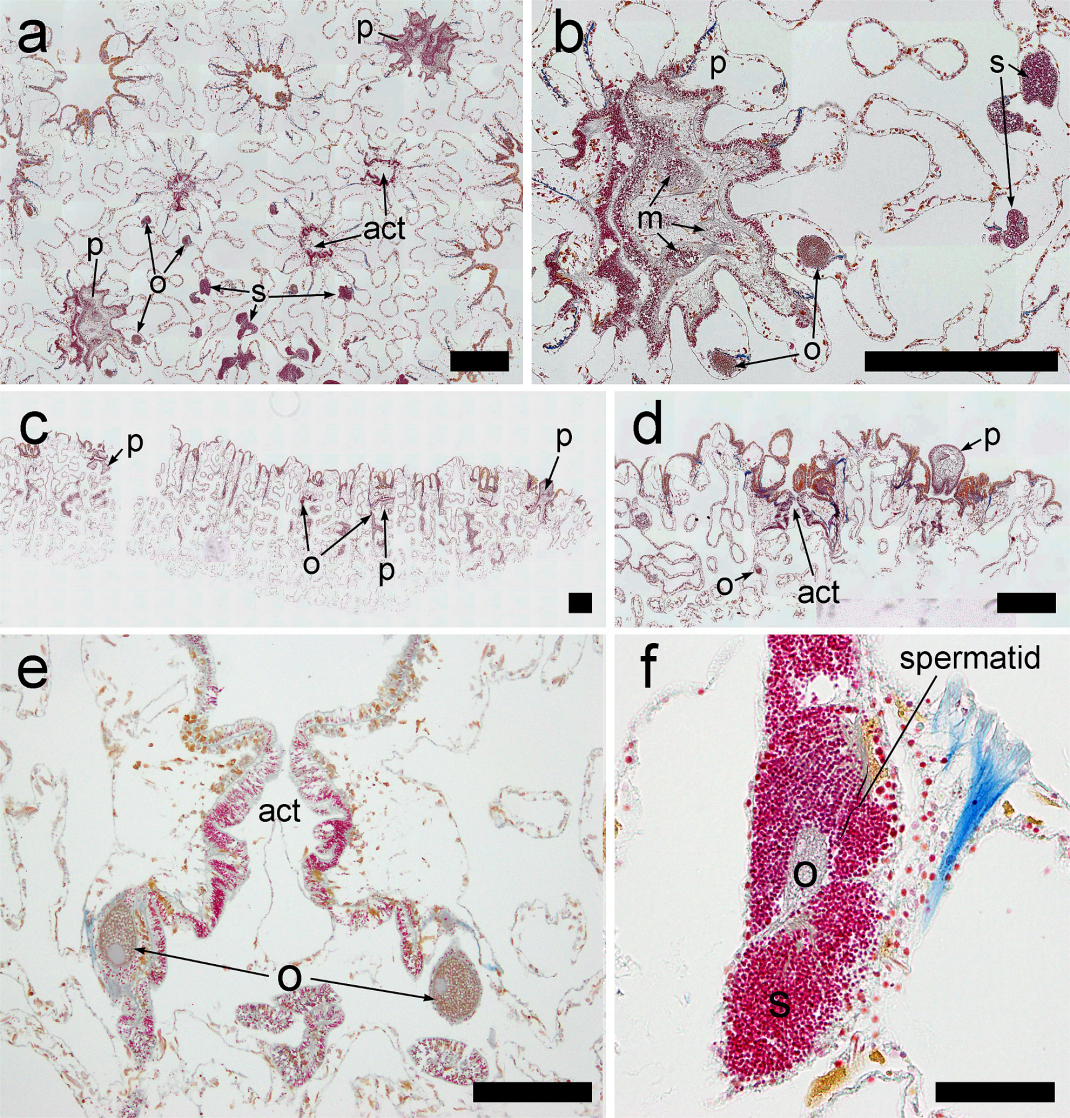
Selected views of reproductive structures of *P*. *astreoides*. (a) Cross-sectional view showing the simultaneous presence of oocytes, spermaries and planulae larvae. The actinopharynx of two polyps is also shown for reference. (b) A close-up view of (a), showing internal details of a larva, including mesenteries. (c) Longitudinal view showing the simultaneous presence of larvae and oocytes. (d) A close-up longitudinal view, showing a larva extruded from the oral opening of a polyp. (e) A longitudinal view of a single polyp, showing the actinopharynx and stage IV ova. (f) A 40x magnification of a stage V spermary and stage IV oocyte. Spermatozoa are arranged in spermatids within the spermary. (o = oocyte/ovum; s = spermary; p = planulae larva; act = actinopharynx; m = mesenteries; bar = 500 μm [a-d], 200 μm [e], and 50 μm [f]).

**Fig 3:**
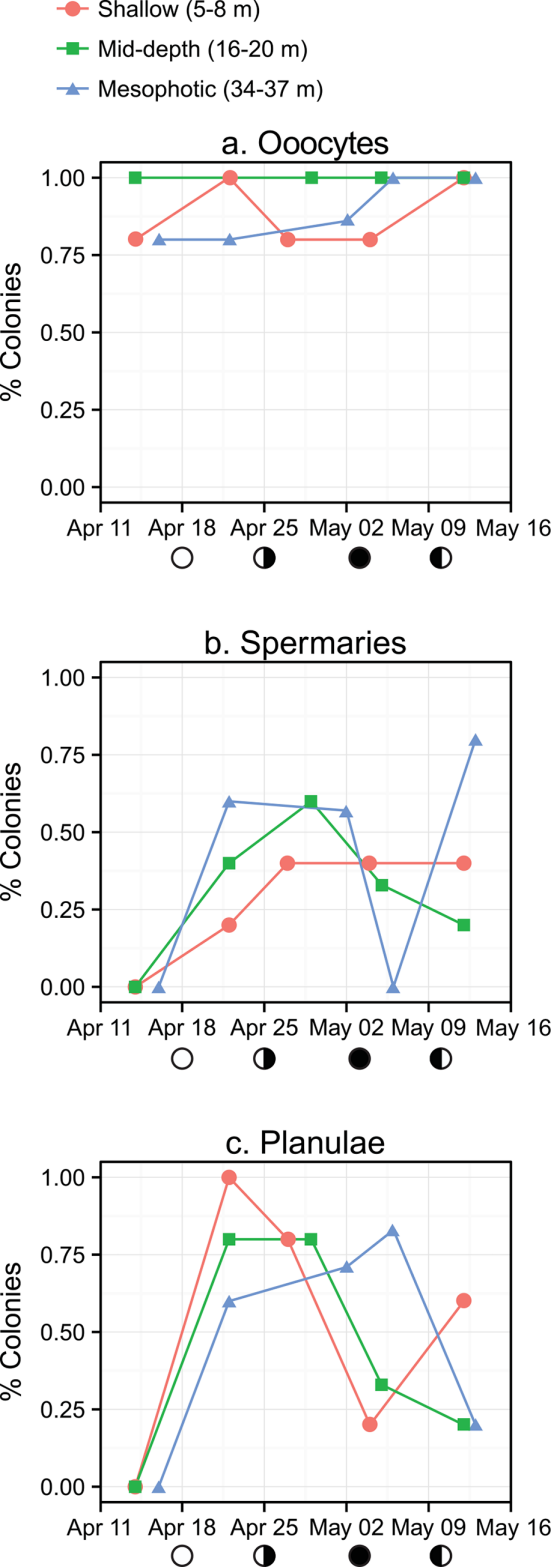
Frequency of *P*. *astreoides* reproductive products found in histological analyses. Values plotted represent the percent of total colonies (3 sites, 5 sampling days, n ≈ 5, N = 76) containing (a) oocytes/ova, (b) spermaries and (c) planulae on each sampling day at each site. The lunar cycle is shown below the x-axis.

Histological analysis of *P*. *astreoides* tissues from all sites revealed the presence of oocytes/ova, spermaries and planulae (Fig 2). The presence of oocytes in tissues was nearly ubiquitous across the five-week sampling period at all sites, and remained present in 80–100% of colonies (Fig 3). The presence of spermaries increased at all sites over the first 2–3 weeks of sampling. Spermaries began to disappear from the tissues of corals from both mid-depth and mesophotic sites after the third week of sampling, which corresponded generally with new moon and is indicative of sperm release. After week 3 spermaries were present in 40% of shallow colonies for the remainder of the sampling period. The presence of spermaries increased dramatically between the fourth and fifth weeks of sampling in mesophotic colonies, indicating that these colonies likely released sperm in May.

**Fig 4:**
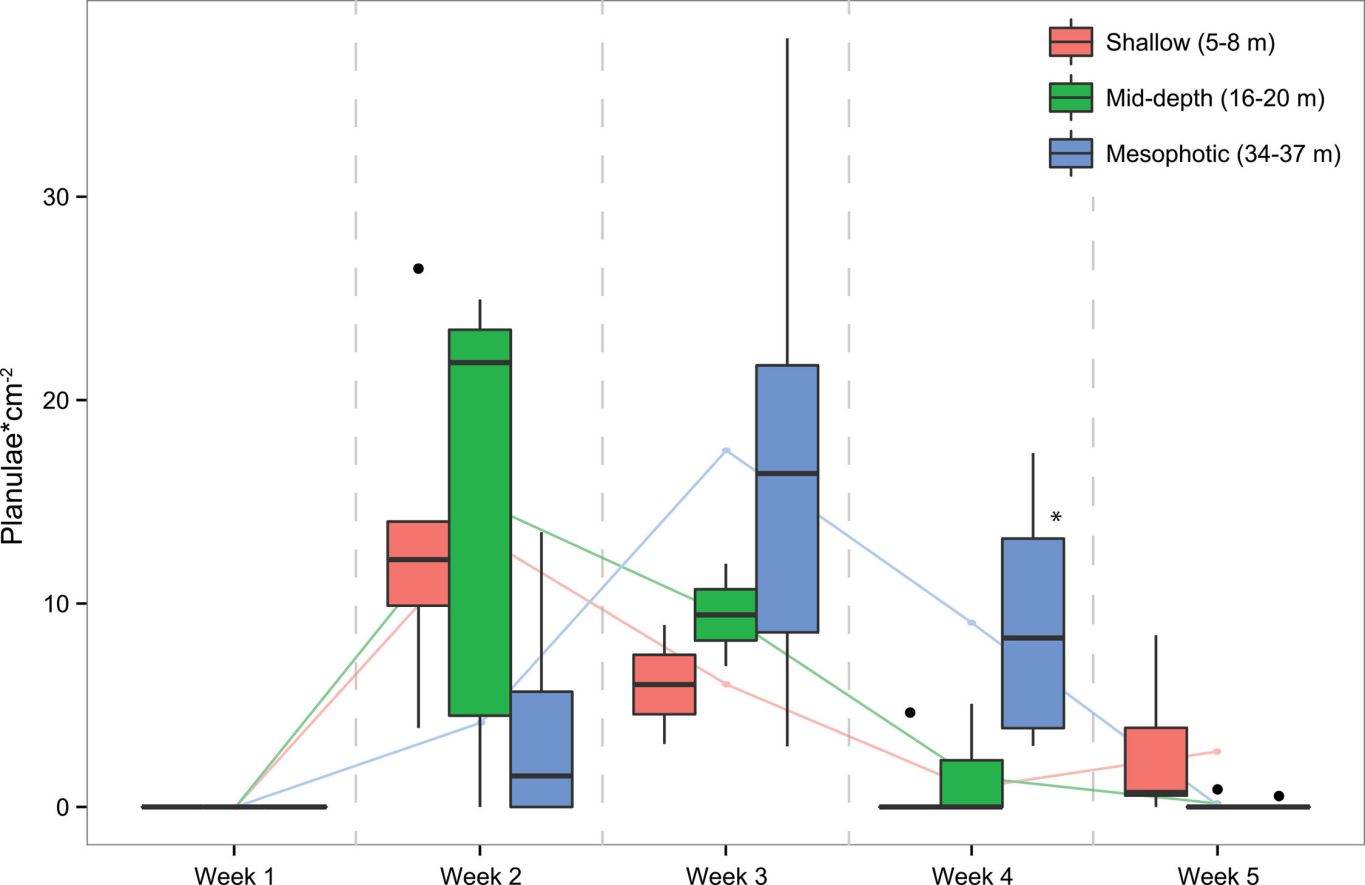
Planulae densities in *P*. *astreoides* tissues. Shallow, mid-depth, and mesophotic planulae densities each week. Upper and lower hinges correspond to the first and third quartiles, bars correspond to medians and whiskers extend to the highest and lowest values within 1.5 times the inter-quartile range. Outliers are represented as dots. Lines connect mean values, and are included to illustrate increasing planulae density, and the loss of those planulae from tissues, presumably due to planulation. At only one week (week 4) were planulae densities found to be significantly different between sites, with higher densities in mesophotic colonies (one-way ANOVA/Tukey’s-HSD, p = 0.009).

The presence of planulae in coral tissues increased over the first two weeks of sampling at all sites, with 100% and 80% of shallow and mid-depth colonies containing planulae by week 2, respectively (Fig 3). Week 2 also corresponded with peak planulae density per cm^2^ in both shallow and mid-depth colonies (Fig 4). Peak in % colonies containing planulae in mesophotic corals did not occur until week 4, however peak planulae density per cm^2^ occurred a week earlier at week 3 (Fig 4). Peak mean planulae density (±SE) was 13.29 cm^-2^ (±3.71), 14.95 cm^-2^ (±5.26) and 17.49 cm^-2^ (±6.00) at shallow, mid-depth and mesophotic sites, respectively, and were not significantly different despite an increasing trend with depth (one-way ANOVA, p > 0.05). The overall average peak planulae density pooled from all sites was found to be 15.24 cm^-2^ (±2.66).

There was a significant difference in planulae density between sites at week 4 (Fig 4). Mesophotic corals had significantly higher average planulae per cm^2^ than shallow or mid-depth corals, which contained near zero planulae (one-way ANOVA/Tukey’s-HSD, p = 0.009). At week 5 planulae density had begun to increase in shallow corals, and not in mid-depth or mesophotic corals, however this difference was not significant.

### Reproductive capability

**Fig 5:**
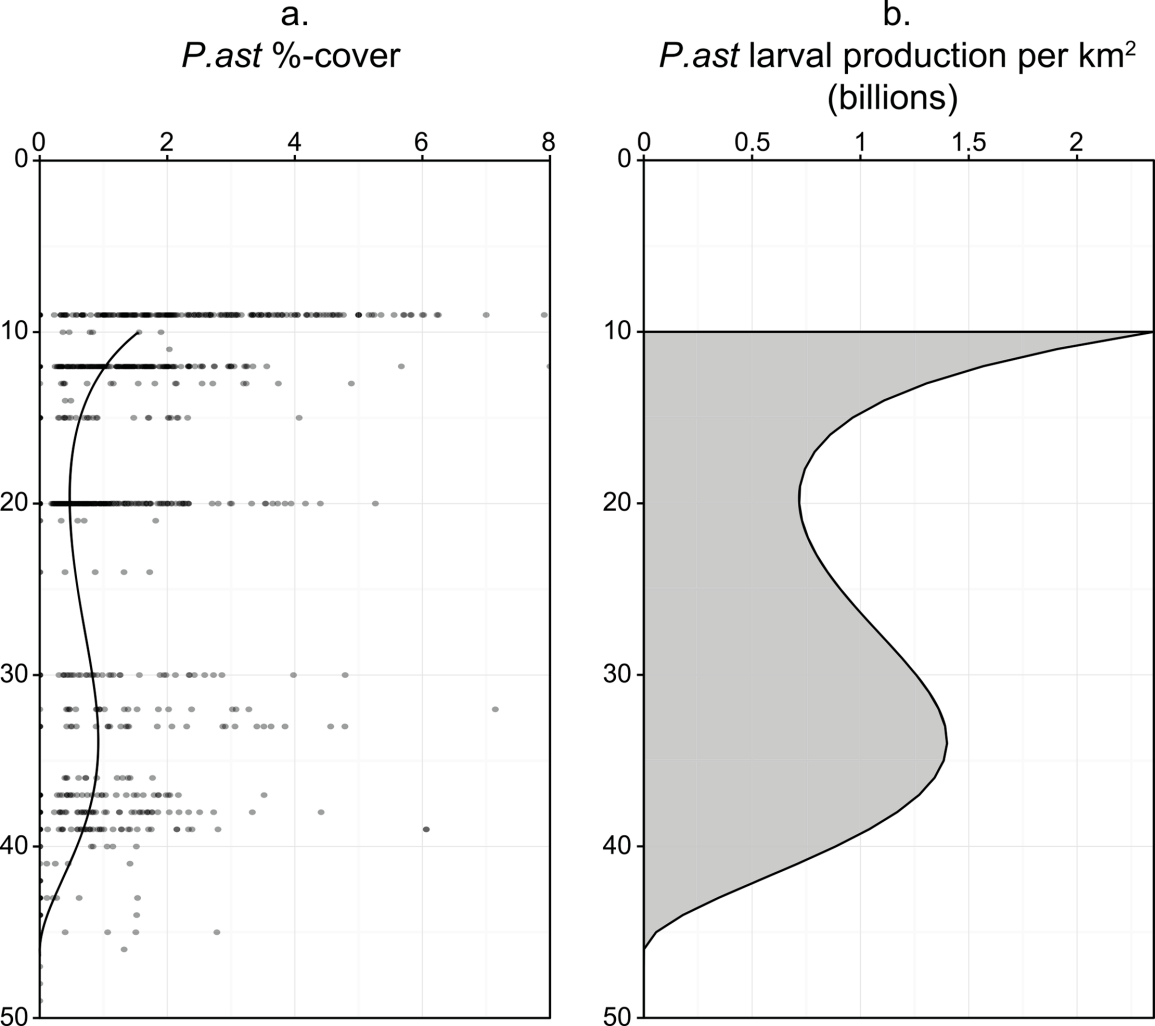
Predictions of planulae production over depth. (a) Relationship of depth and cover of *P*. *astreoides* at sites south of St. Thomas, USVI derived from the Territorial Coral Reef Monitoring Program [30] from 2001–2012. (b) Predicted number of larvae produced in a hypothetical 1 km^2^ coral reef over depth, calculated as the products of planulae per cm^2^ and coral cover.

*Porites astreoides* coral cover estimates were pooled from coral reef monitoring surveys (2001–2012), plotted against depth, and a third-order polynomial was fitted to the data (p < 0.0001, R^2^ = 0.20) (Fig 5). In this model coral cover of *P*. *astreoides* is relatively consistent across depth, with means ranging from ~0.5–1.5%, and an apparent dip in % cover between 15 and 25m. By 50m depth *P*. *astreoides* coral cover drops to near zero. The resulting regression line was used in conjunction with planulae density estimates (fecundity) to estimate *P*. *astreoides* planulae production over a hypothetical 1 km^2^ reef from 10–50 m depth, based on equal planulae production (15.24 per cm^2^) (Fig 5). Because fecundity was equal at all depths, estimates of depth-specific planulae production were entirely dependent on coral cover. High coral cover shallower than 15 m results in nearly 1.5 billion (300%) more planulae produced per km^2^ at 10 m versus at 20 m. A deep peak in coral cover between 30 m and 35 m suggests nearly double planulae production at these depths compared to 20 m, however production at 10 m is estimated to be nearly 40% higher than at 35 m.

### Aquarium planulation observations

Shallow colonies measured approximately 238 cm^2^, 146 cm^2^, and 264 cm^2^. Mid-depth colonies measured approximately 126 cm^2^, 101 cm^2^, and 251 cm^2^. Mesophotic colonies measured approximately 213 cm^2^, 118 cm^2^, and 507 cm^2^. Aquarium observations were complicated by less than ideal water conditions, and thus results are anecdotal. Shallow and mid-depth colonies showed signs of stress including paling and shedding mucous, likely due to high water temperature. Mesophotic colonies that were collected several days later did not show signs of stress on the nights of observation. One mid-depth colony (251 cm^2^) planulated during observations, and released between 50–100 larvae on the evening of May 3^rd^. No planulation was observed from shallow colonies. All mesophotic colonies planulated in the laboratory over the two evenings of observation. These colonies released 1000s of larvae each over the two nights of observation.

## Discussion

*Porites astreoides* colonies sampled from mesophotic reefs in the US Virgin Islands were found to reproduce in a similar gametogenic cycle with and have similar fecundity to shallower conspecifics. This suggests that depth may provide a reproductive refuge for this species by providing habitat conducive to coral reproduction that is removed from shallow and coastal coral stressors. Evidence provided in this study adds credit to the argument that MCEs could serve as refugia habitat for these species as well, by providing stable habitat over evolutionary time scales from which populations could expand following climatic change [2,31].

Although we did not observe shallow *P*. *astreoides* planulation in the laboratory, mesophotic colonies (and one mid-depth colony) planulated on evenings predicted for shallow colonies in the Caribbean [25–27]. Histological evidence suggests that the date at which peak larval density in colony tissues occurs may be delayed by as much as a week in USVI mesophotic colonies as compared to shallow and mid-depth colonies, and a similar delay was seen in mesophotic colony planulation, as indicated by a loss of planulae from coral tissues. It is possible that USVI mesophotic *P*. *astreoides* experience environmental conditions that alter reproductive physiology or cues; however, there is high variability in the dates of planulation in this species, with a range in planulation of more than 20 days straddling the new moon [27]. Thus, the delayed planulation in mesophotic colonies may not be due to depth, but it may indicate distinct breeding populations. The signal of sperm release, or spawn, was much clearer in mesophotic colonies, as indicated by the loss of sperm and spermaries from tissues. A subsequent increase in the presence of spermaries on the final week of sampling likely indicates continued energetic investment in reproduction, and spawning in the following month.

It is unlikely that direct interbreeding occurs between mesophotic and shallow colonies of *P*. *astreoides* in the USVI, despite the potential for synchronous gametogenesis and spawning behavior, because these habitats are separated by considerable horizontal distances in most cases. However, interbreeding would be more likely on wall or seamount habitats, where swimming sperm may have a greater potential of fertilization with conspecifics from a wide range of depths.

No differences were seen over depth in the peak density of larvae in *P*. *astreoides* tissues, which implies that depth and reduced photosynthectically available radiation encountered at upper mesophotic depths do not negatively effect the production of planulae larvae in this species. In fact, a non-significant but increasing trend was observed in peak planulae density over depth, with highest larval density occurring at mesophotic depth. Two peaks in larval production were predicted at 10 m and ~35 m, due to peaks in *P*. *astreoides* coral cover. These results are an interesting contrast to those found for populations of the broadcast spawning coral *O*. *faveolata* in the USVI, which increase in both abundance and fecundity with depth [8]. Although depth appears to provide a reproductive refuge for *P*. *astreoides* in the USVI, the effect is not so pronounced as it is for *O*. *faveolata*. It should be noted that to estimate reproductive output several assumptions were made that may require further study. The model used assumes equal fecundity over all *P*. *astreoides* coral surface area, and similar size-class distributions at all sites/depths. Fecundity may increase with size in *P*. *astreoides* [26], and if size-class distribution changes with depth, the assumption of equal fecundity over all coral surface area may not hold.

There are several potential ways in which mesophotic *P*. *astreoides* colonies could maintain reproductive output despite reduced photosynthetically available radiation. The first is that the energy required for reproduction in this species is not limited by depth, and decoupled from light saturation [8]. Bimodal peaks in coral growth have been observed in shallow and mesophotic habitats (*Madracis mirabilis*), and deep peaks in growth have been attributed to increased heterotrophy below the summer deep chlorophyll maximum layer [32]. MCEs in the USVI are associated with the deep chlorophyll maximum layer, which may result in increased advective food supply [4].

Reduced disturbance in mesophotic environments [4–6,11,33,34] may similarly reduce metabolic demands on USVI mesophotic *P*. *astreoides*, allowing them to divert energy otherwise allocated to growth and tissue maintenance in shallow corals to reproduction [8,35]. In direct contrast, it is possible that adverse conditions in USVI MCEs increase adaptive pressure on successful gametogenesis and reproduction in *P*. *astreoides*, as can occur in plants at the edges of their geographic range [36].

It is worthwhile to note that the production of larvae in brooding corals is a product not only of individual coral phenotype, but also of population demography; the density of adult reproductive individuals has implications for the successful fertilization of internal ova. In locations where *P*. *astreoides* is missing or rare at depths across its depth range, the relationship of depth and planulae production would be expected to change accordingly. Thus, the relationship observed in the USVI–where mesophotic cover of *P*. *astreoides* is relatively high–may differ from those in other locations in the Caribbean.

### Implications and knowledge gaps

Deeper living corals may produce lower concentrations of mycosporene-like amino acids (MMAs) that protect coral tissues from damaging ultraviolet light. Several studies have shown that this may result in lower survivorship of brooded coral larvae from deeper populations [37,38]. Reduced pelagic and post-settlement survivorship of mesophotic *P*. *astreoides* larvae moving into shallower environments could limit the effectiveness of depth as a reproductive refuge for shallow populations. However, dispersal and connectivity modeling of *P*. *astreoides* in the USVI suggests a high potential for shorter distance and multigenerational vertical connectivity [7] and a genetic study of *P*. *astreoides* populations has shown high gene flow across depths in the USVI (7–33 m) and in Bermuda (4–26 m) [39]. Thus, despite the high potential for mortality during or after settlement into shallower habitats, mesophotic *P*. *astreoides* larvae are capable of vertical migrations and depth may provide a refugium for this species.

*P*. *astreoides* has been shown to be relatively resistant to isolated high thermal stress events [40], but it has been suggested that *P*. *astreoides* may be particularly vulnerable to repetitive annual coral bleaching [41]. Despite the potential for individual mortality, the species’ life-history and larval traits may make certain connected populations particularly robust to the effects of climate change [7]. In some parts of the Caribbean, recruitment of poritids is much higher than other–mostly broadcast spawning–species [42]. All of this evidence suggests that *P*. *astreoides* is an important coral to continue to monitor and study, because how populations of this coral respond to climate change has important implications for how colony resistance, perturbation, life-history and connectivity affect metapopulation persistence in Caribbean corals.

## Supporting Information

S1 Dataset Supporting Data. Weekly gamete and planulae presence/absence in coral tissues and planulae densities.
(XLSX)
